# Clinicopathological findings of systemic Epstein-Barr virus-positive T-lymphoproliferative diseases in younger and older adults

**DOI:** 10.1186/s13000-021-01107-1

**Published:** 2021-06-04

**Authors:** Ziyao Wang, Shoichi Kimura, Hiromi Iwasaki, Ken Takase, Yumi Oshiro, Ayako Gamachi, Kosuke Makihara, Masao Ogata, Tsutomu Daa, Seiya Momosaki, Yasushi Takamatsu, Morishige Takeshita

**Affiliations:** 1 Graduate School of Medical Sciences, Fukuoka University, 7-45-1 Nanakuma, Jonan-ku, Fukuoka, 8140180 Japan; 2 Department of Pathology, Faculty of Medicine, Fukuoka University, 7-45-1 Nanakuma, Jonan-ku, Fukuoka, 8140180 Japan; 3 Departments of Hematology, Clinical Research Center, National Hospital Organization Kyushu Medical Center, 1-8-1 Jigyohama, Chuo-ku, Fukuoka, 8108563 Japan; 4 Department of Pathology, Matsuyama Red Cross Hospital, 1 Bunkyo-cho, Matsuyama, 7910000 Japan; 5 Department of Pathology, Almeida Memorial Hospital, 1509-2. Oita, Miyazaki, 8701195 Japan; 6 Department of Pathology, Kyushu Rosai Hospital, 1-1, Sonekita, Kokura South Ward, Kitakyushu, 800-0296 Japan; 7 Departments of Hematology, Faculty of Medicine, Oita University, Idaigaoka, Hazama-machi, Yufushi, Oita, 8795593 Japan; 8 Departments of Pathology, Faculty of Medicine, Oita University, Idaigaoka, Hazama-machi, Yufushi, Oita, 8795593 Japan; 9 Departments of Pathology, Clinical Research Center, National Hospital Organization Kyushu Medical Center, 1-8-1 Jigyohama, Chuo-ku, Fukuoka, 8108563 Japan; 10 Departments of Internal Medicine, Division of Medical Oncology, Hematology and Infectious Disease, Faculty of Medicine, and Fukuoka University, 7-45-1 Nanakuma, Jonan-ku, Fukuoka, 8140180 Japan

**Keywords:** Epstein-Barr virus, Systemic EBV^+^ T-cell lymphoma of childhood, CD8^+^ T-cell lymphoma, Hemophagocytic lymphohistiocytosis

## Abstract

**Background:**

Systemic Epstein-Barr virus^+^ T-cell lymphoma (sEBV^+^ TCL) occurs in childhood and young adults, and is exceptionally rare in older adults.

**Methods:**

We investigated clinicopathological features in 16 patients of various ages with systemic EBV^+^ CD8^+^ T-lymphoproliferative diseases.

**Results:**

Eight younger patients and four of eight older adults had sEBV^+^ CD8^+^ TCL, with invasion by medium-sized to/or large atypical lymphocytes primarily in bone marrow and lymph nodes, hemophagocytic lymphohistiocytosis (HLH), and progressive clinicopathological course. A further two patients demonstrated EBV^+^ node-based CD8^+^ large TCL without HLH, while the remaining two had the systemic form of chronic active EBV infection (sCAEBV) with CD8^+^ small lymphocytes. Past history of sCAEBV-like lesions was observed in one sEBV^+^ TCL patient (8.3%). Immunohistologically, in 12 sEBV^+^ TCL patients, atypical lymphocytes were positive for phosphate signal transducer and activator of transcription 3 (66.7%), CMYC (83.3%), and p53 (75%). Strong reactions of programmed cell death-ligand (PD-L)1^+^ tumor or non-neoplastic cells were detected in nine sEBV^+^ TCL patients (75%). Clonal peaks of the T-cell receptor (*TCR) γ* gene were detected in eight sEBV^+^ TCL patients by polymerase chain reaction. Four younger patients in sEBV^+^ TCL (33.3%) are in remission with chemotherapies including etoposide, and three of the four underwent allogeneic stem cell transplantation (SCT).

**Conclusion:**

sEBV^+^ CD8^+^ TCL was observed in younger and older adults with less history of sCAEBV. HLH, tumor cell atypia, immunohistological findings, and progressive clinical course were characteristic of sEBV^+^ CD8^+^ TCL. Prompt chemotherapy and SCT induced tumor regression in sEBV^+^ CD8^+^ TCL patients.

## Background

Epstein-Barr virus (EBV)-positive T/natural killer (NK)-cell lymphoproliferative diseases (EBV^+^ TNKLPDs) with bone marrow involvement include aggressive NK-cell leukemia, hemophagocytic lymphohistiocytosis (HLH), and systemic EBV^+^ T-cell lymphoma (sEBV^+^ TCL) of childhood [1–3]. Patients with these TNKLPDs exhibit similar clinicopathological features of high fever, hepatosplenomegaly, pan- or bicytopenia, and coagulopathy. EBV^+^ HLH patients occur predominantly in infants and young adults, and showed increased CD8^+^ T-cells and small amounts of EBV^+^ cells in bone marrow. The HLH patients frequently recover following treatments with the HLH-2004 regimen including etoposide (VP16) and cyclosporine A, and hematopoietic stem cell transplantation (SCT) [4–6]. In contrast, sEBV^+^ TCL of childhood is primarily characterized by EBV^+^ CD8^+^ and CD56^−^ atypical T-cells in bone marrow, lymph nodes, liver, and spleen, and patients demonstrate above described clinical features of TNKLPDs and mostly follow a rapidly progressive clinical course. Several patients with sEBV^+^ TCL of childhood were reported to have past history of the systemic form of chronic active EBV (sCAEBV) infection [1, 7, 8]. Patients with sCAEBV reveal persistent infectious mononucleosis (IM)-like symptoms, primarily increased EBV^+^ CD4^+^, occasionally CD8^+^ T-cells and CD56^+^ NK-cells [9]. sEBV^+^ TCL has previously been confused with lethal HLH and sCAEBV [2, 9, 10]. Additionally, EBV^+^ nodal (node-based) cytotoxic T/NK-cell lymphoma patients showed frequent hepatic and systemic tumor invasions without features of HLH, and followed a progressive clinical course [11, 12]. Differentiation between sEBV^+^ and EBV^+^ nodal CD8^+^ TCL was also difficult.

EBV+ CD8+ T lymphoproliferative diseases (TLPDs) are exceptionally rare. In the present study, we investigated 16 patients with systemic EBV^+^ CD8^+^ TLPDs with involvements in bone marrow, lymph nodes, and liver. Among them, eight were over 50 years old. We primarily present clinicopathological findings from 12 sEBV^+^ CD8^+^ TCL patients in childhood and older age, and compared them with those from EBV^+^ CD8^+^ nodal TCL and sCAEBV with CD8^+^ T-cells.

## Materials and methods

### Patient selection and clinical findings

Registered patients were retrieved retrospectively over the period from 1990 to 2020 from the Department of Pathology, Fukuoka University. Histological classifications were performed based on the WHO classification (2017) and detailed descriptions of EBV^+^ TNKLPDs [2, 10, 13]. Criteria for sEBV^+^ TCL of childhood were as follows: progressive clinical course with features of HLH, overt infiltrates of CD8^+^ and CD56^−^ cytotoxic T-lymphocytes with small or occasionally medium to large-sized or large nuclei, and overt lymphoma or rearrangements of T-cell receptor (*TCR*) genes. EBV^+^ nodal cytotoxic TCL shows a primarily nodal presentation with limited extranodal disease and no nasal invasion, mainly demonstrating CD8^+^ and CD56-negative phenotype [11, 12]. sCAEBV presents mainly with IM-like symptoms persisting for 3 months, elevated peripheral blood EBV DNA (≥ 1 × 10^2.5^ copies/μg) and the presence of EBV^+^ T-lymphocytes with mild nuclear atypia [9, 14]. In this study, EBV^+^ HLH was excluded because of small amounts of EBV-encoded RNAs (EBERs)-positive cells [15], and we excluded CD8^+^ and CD56^+^ EBV^+^ nasal type TNKCL [2, 3]. Laboratory criteria for cytopenia, coagulopathy, serum ferritin and sIL2R referred to those of HLH [13].

### Histology, immunohistology, and detection of EBV-encoded RNA

Excised tissue specimens were fixed in 10% formalin to generate formalin-fixed paraffin-embedded (FFPE) samples, and were stained with hematoxylin and eosin. Bone marrow clot and biopsy samples, and related smears were examined in all 16 patients. Samples from involved lymph nodes, liver, and other organs were also examined. Autopsy examination was permitted in three older sEBV^+^ TCL patients. For immunohistochemistry, antibodies were applied to formalin-fixed tumor samples using a Leica Bond III-automated stainer (Leica Biosystems, Buffalo Grove, IL), and peroxidase reaction was developed using diaminobenzidine. The following antibodies were used: CD3 (PS1, Leica, Newcastle, UK), CD4 (4B12, Leica), CD8 (C81/44B, Leica), TIA1 (2GP, Beckman Coulter, Marseille, France), granzyme B (11F1, Leica), CD56 (1B6, Leica), TCRβF1 (8A3, Endogen, Rockford, IL), TCRCγM1 (γ3.20, Endogen), phosphate signal transducer and activator of transcription 3 (pSTAT3) (RB5791, Argent, San Diego, CA), CD30 (BerH2, Dako, Glostrup, Denmark), programmed cell death 1 (PD1) (NAT105, Abcam, Cambridge, MA), PD-ligand 1 (PD-L1) (E1L3N, Cell Signaling, Danvers, MA), CMYC (Y69, Abcam), p53 (DO7, Leica), Chemokine receptor (CXCR) 3 (1C6, Bioscience, San Diego, CA), CC chemokine receptor (CCR) 4 (1G1, Bioscience), CD20 (L26, Nichirei, Tokyo), latent membrane protein (LMP) 1 (CS1–4, Dako), and EBV nuclear antigen (EBNA) 2 (PE2, Leica). For all immunostains, reactions were considered positive when over 30% of tumor cells stained positively. Among them, ≥50% of tumor cells stained positively for CMYC, p53, and PD-L1 were considered positive. Staining intensity of PD-L1 for non-neoplastic cells including histiocytes and dendritic cells, were scored as: R0 (no positive cells/HPF), R1^+^ (a few to 5% cells), R2^+^ (≥ 5 to 20%) and R3^+^ (≥ 20%) [16]. The presence of EBV infection was determined by in situ hybridization of EBERs^+^ nuclear signals in over 50% of atypical lymphoid cells. For EBERs stains, deparaffinized tissue sections were hybridized in a solution of 50% formamide containing fluorescein isothiocyanate-labeled EBERs oligonucleotides (EBERs probe, Leica). Double staining of lymphocyte markers and EBERs was performed to confirm the presence of EBV^+^ CD8^+^ T-cells.

### Molecular analysis of the *TCR*γ gene locus

For polymerase chain reaction (PCR) amplification of the *TCR*γ locus, DNA was prepared from FFPE samples of 11 available patients by standard proteinase K digestion and phenol-chloroform extraction. BIOMED-2 multiplex PCR assays (InVivoScribe Technologies, San Diego, CA) of *TCR*γ (Bottles A, B; V-J and D-J gene rearrangements) were performed using standardized protocols and primers [17]. Following amplification, *TCR*γ-PCR products were analyzed using GeneScan (MultiNA, Shimazu Co., Kyoto, Japan). Monoclonality was defined as single or two predominant PCR peaks. The PCR peaks five-fold higher than the third highest PCR peak were estimated as monoclonality.

## Results

### Clinical findings

Baseline clinical features, treatments, and prognoses of the 16 patients with EBV^+^ CD8^+^ TLPDs are shown in Table 1. Patient Nos. 1–12 were ultimately diagnosed with sEBV^+^ TCL, Nos. 13 and 14 with EBV^+^ nodal TCL, and Nos. 15 and 16 with sCAEBV. The median age of the 12 sEBV^+^ TCL patients was 36.1 years, four of whom were over 60 years of age. Fourteen patients were previously healthy without previously documented immunosuppressive states and malignant disease. Patient No. 12 received operation for thyroid cancer before 20 years, and patient No 16 was a hepatitis B virus carrier. One younger sEBV^+^ TCL patient (No. 7) was pregnant, while an older adult (No. 9) had past history of cutaneous CAEBV-like lesions 6 months before overt manifestation. High fever (*n* = 12), lymphadenopathy (*n* = 9) and hepatosplenomegaly (*n* = 10) were observed in a short period of time in sEBV^+^ TCL patients. Bi- or pancytopenia was detected in all 12 patients with sEBV^+^ TCL, and hyperferritinemia (> 500 μg/L) was detected in 10. Hyperbilirubinemia (≥2.0 mg/dL) was detected in seven sEBV^+^ TCL patients, and both elevated liver enzymes and lactate dehydrogenase (LDH) (≥300 U/L) were detected in all 12. Elevated sIL2R (≥2400 U/mL) was observed in nine sEBV^+^ TCL patients. Examination of five younger sEBV^+^ TCL patients revealed elevated EBV DNA copies (> 1 × 10^2.5^ copies/μg) in peripheral blood. An acute EBV infection pattern was detected in two younger sEBV^+^ TCL patients by examination of viral capsid antigens IgM/IgG and EBNA IgG, and a past infection pattern was detected in eight with sEBV^+^ TCL. Two EBV^+^ nodal TCL patients had high fever, systemic lymphadenopathy and elevated LDH, but were negative for thrombocytopenia and liver dysfunction. Patient No. 14 demonstrated bicytopenia and hyperferritinemia, and had past history of erythematous skin lesions 10 months before overt manifestations. Two patients with sCAEBV demonstrated high fever, IM-like symptoms, pancytopenia, high LDH and sIL2R titers, and elevated EBV DNA copies in peripheral blood. Patient No. 15 had no features of HLH, but patient No. 16 exhibited HLH. Patient No.15 had past history of liver dysfunction 24 months before overt manifestations. All 16 patients were classified as clinical stage 4.

**Table 1:** Clinical data, treatment and prognosis of systemic and nodal EBV^+^ T-cell lymphoma and systemic form of chronic active EBV infection

Patient No.	Age	Sex	Final Diagnosis	Lymphadenopathy	Hepatosplenomegaly	Cytopenia	Coagulopathy	Ferritin (ng/ml)	TB (mg/dL)	LDH (U/L)	sIL2R (U/ml)	EBV DNA	VCA IgG/M	Treatments	Follow up (months)	Outcome
1	2	M	sEBV^+^ TCL	+	+	pan-cytopenia	+ (DIC)	1492	0.7	3876	9450	nt	+/-	γ-globulin, Dexa	2	dead
2	3	M	sEBV^+^ TCL	+	+	pan-cytopenia	+	8427	1.4	1488	15043	6x10^4^	+/+	VP16, CHOP, ASCT	48	alive
3	19	F	sEBV^+^ TCL	+	+	bi-cytopenia	+	1547	2.7	715	nt	2.5x10^5^	+/-	CsA, VP16, CHOP	3	dead
4	21	M	sEBV^+^ TCL	-	+	pan-cytopenia	+ (DIC)	17598	15	1173	49646	1.9x10^5^	+/+	VP16, CHOP	3	dead
5	23	F	sEBV^+^ TCL	+	+	pan-cytopenia	+	1950	0.5	873	1963	1.8x10^5^	+/-	CsA, CHOP	20	alive
6	24	M	sEBV^+^ TCL	-	+	bi-cytopenia	+ (DIC)	161345	3.7	1300	9170	1.4x10^5^	+/-	HLH-2004, SMILE, ASCT	15	alive
7	30	F	sEBV^+^ TCL	-	+	pan-cytopenia	+ (DIC)	216	5.2	854	nt	nt	+/-	Dexa	1	dead
8	36	M	sEBV^+^ TCL	+	-	bi-cytopenia	+	1802	0.4	3874	21100	nt	+/-	VP16, CHOP, ASCT	18	alive
9	64	F	sEBV^+^ TCL	+	-	bi-cytopenia	+	4990	0.8	2009	13101	nt	nt	CHOP, MTX	4	dead
10	65	M	sEBV^+^ TCL	+	+	pan-cytopenia	+ (DIC)	12169	10	1538	5530	nt	+/-	CHOP	1	dead
11	70	M	sEBV^+^ TCL	+	+	bi-cytopenia	+	no	3.2	1281	11268	nt	nt	DeVIC	1	dead
12	76	F	sEBV^+^ TCL	+	+	pan-cytopenia	+ (DIC)	13325	7.1	2606	23234	nt	+/-	Dexa	1	dead
13	62	M	EBV^+^ nodal TCL	+	-	normal	-	nt	0.4	513	1474	nt	nt	CHOP	7	dead
14	81	F	EBV^+^ nodal TCL	+	-	bi-cytopenia	-	2810	0.9	907	4878	nt	nt	CHOP	85	alive
15	54	F	CAEBV	+	+	pan-cytopenia	-	537	1.5	788	3205	1.5x10^5^	+/-	R-CHOP, CsA, Capizzi	4	dead
16	65	M	CAEBV	+	+	pan-cytopenia	+ (DIC)	5455	9.6	781	11800	1.2x10^5^	+/-	Dexa, VP16	21	alive

Autopsy examination was performed in patients No. 10, 11, 12

*CAEBV* chronic active EBV, *DIC* disseminated intravascular coagulation, *nt* no test, *TB* total bilirubin, *TCL* T-cell lymphoma, *VCA* viral capsid antigen, *ASCT* allogeneic stem cell transplantation, *Capizzi* cytosine arabinoside, L-asparaginase, predonine, MTX, *CHOP* cyclophosphamide, doxorubicin, vincristine, prednisone, *CsA* cyclosporine A, *DeVIC* Dexa, VP16, ifosfamide, carboplatin, *Dexa* dexamethasone, *HLH-2004* Dexa, VP16, CsA, *MTX* methotrexate, *SMILE* Dexa, MTX, ifosfamide, VP16, L-asparaginase

### Pathological and immunohistological findings

**Fig. 1:**
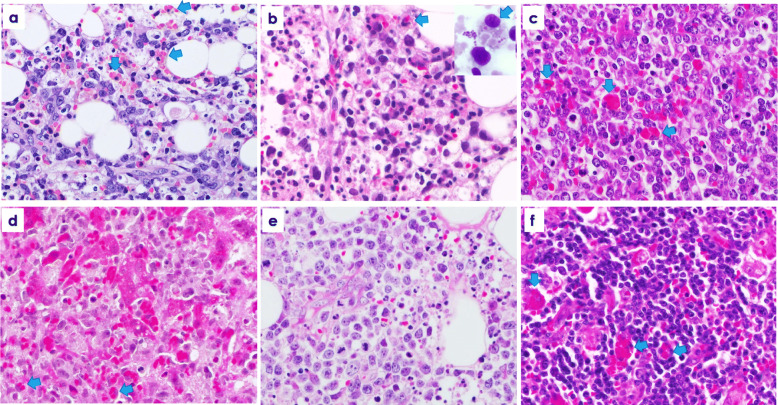
**a** Diffuse infiltrate of large atypical lymphocytes and a few erythrophagocytes (arrow) in bone marrow (patient No. 4). **b** Bone marrow invasion by atypical medium-sized and some large atypical lymphocytes with hyperchromatic nuclei in patient No. 12. Histiocytic reaction and a few erythrophagocytes were detected. An erythrophagocyte and atypical lymphocyte in a smear. **c** Lymph node involvement of large atypical lymphocytes and some erythrophagocytes in patient No. 5. **d** Diffuse infiltrates of large atypical lymphocytes in the hepatic lobules (patient No. 11). Destructed hepatic trabeculae are observable in the right upper part, and a few erythrophagocytes are seen. **e** Diffuse infiltrate of large atypical lymphocytes and many apoptotic bodies in involved subcutaneous tissue of patient No. 13. **f** Diffuse infiltrate of small lymphocytes with mild atypia and several scattered erythrophagocytes in lymph node of patient No. 16. × 400

Histological and immunohistological findings in involved organs are shown in Table 2. Scattered and diffuse infiltrates of atypical medium-sized to/or large lymphocytes were detected in bone marrow of 10 sEBV^+^ TCL patients, and partial infiltrates of atypical lymphocytes were observed in two older adults (Nos. 10 and 12). Increased histiocytes and hemophagocytic macrophages were detected in bone marrow and related smears in 12 sEBV^+^ TCL patients (Fig. 1a, b). Diffuse infiltrates of pleomorphic medium-sized to/or large lymphocytes were found in lymph nodes in three sEBV^+^ TCL patients, and in pleura and liver in one patient each (Fig. 1c, d). Two EBV^+^ nodal TCL patients demonstrated diffuse invasion by large atypical lymphocytes in lymph nodes and subcutis (Fig. 1e). Focal bone marrow invasion by atypical large lymphocytes was found in one EBV^+^ nodal TCL patient (No. 14), although no hemophagocytosis was observed in two. Two patients with CAEBV showed diffuse infiltrates of small atypical lymphocytes in lymph nodes (Fig. 1f). Patient No. 15 showed patchy infiltrates of small lymphocytes in the hepatic sinuses and portal areas of the liver, but not in the bone marrow.

**Table 2:** Pathology, immunohistology, and *TCRγ* gene of systemic and nodal EBV^+^ T-cell lymphoma and systemic form of chronic active EBV infection

Patient No.	BM involvement	Hemophagocytosis	Examined tissues	Tumor cell size	CD3	TCR βF1	CD8	CTG	CXCR3	pSTAT3	CMYC (%)	p53 (%)	PD-L1	LMP1/ EBNA2	EBER	*TCRγ* gene
1	+	+	LN	med, large	+	+	+	+	+	-	10	40	R3+	+/-	+	C
2	+	+	BM	med, large	+	-	+	+	-	+	50	50	R3+	-/-	+	nt
3	+	+	BM	med, large	+	-	+	+	+	+	70	30	R1+	-/-	+	C
4	+	+	BM	large	+	+	+	+	+	+	70	70	R1+	-/-	+	C
5	+	+	LN	large	+	+	+	+	+	-	50	70	R3+	-/-	+	nt
6	+	+	BM	large	+	+	+	+	-	+	70	80	R2+	-/-	+	C
7	+	+	BM	med, large	+	-	+	+	+	+	60	50	R3+^b^	-/-	+	C
8	+	+	BM	large	+	-	+	+	+	+	70	40	R1+	+/-	+	nt
9	+	+	LN	med, large	+	+	+	+	+	-	40	50	R3+	+/-	+	C
10	+^a^	+	BM, Pleura	large	+	+	+	+	+	+	80	50	R3+	-/-	+	C
11	+	+	Liver	large	+	-	+	+	+	-	50	60	R3+	-/-	+	C
12	+^a^	+	BM	med, large	+	-	+	+	+	+	50	60	R2+	-/-	+	nt
13	-	-	LN, SC	large	+	-	+	+	-	+	70	90	R2+	+/-	+	C
14	+^a^	-	LN	large	+	+	+	+	+	+	70	40	R3+^b^	+/-	+	C
15	-	-	LN, Liver	small	+	-	+	+	-	-	10	40	R1+	-/-	+	poly
16	+	+	LN	small	+	+	+	+	+	+	10	20	R3+	-/-	+	nt

*BM* bone marrow, *C* clonal peaks, *CTG* positive for TIA1 and/or Granzyme B, *LN* lymph node, *med* medium-sized, *nt* no test, *R* Reaction, *SC* subcutis

^a^Partial infiltrate of atypical CD8+ EBV+ lymphocytes is found. ^b^Tumor cells are also positive for PD-L1

**Fig. 2:**
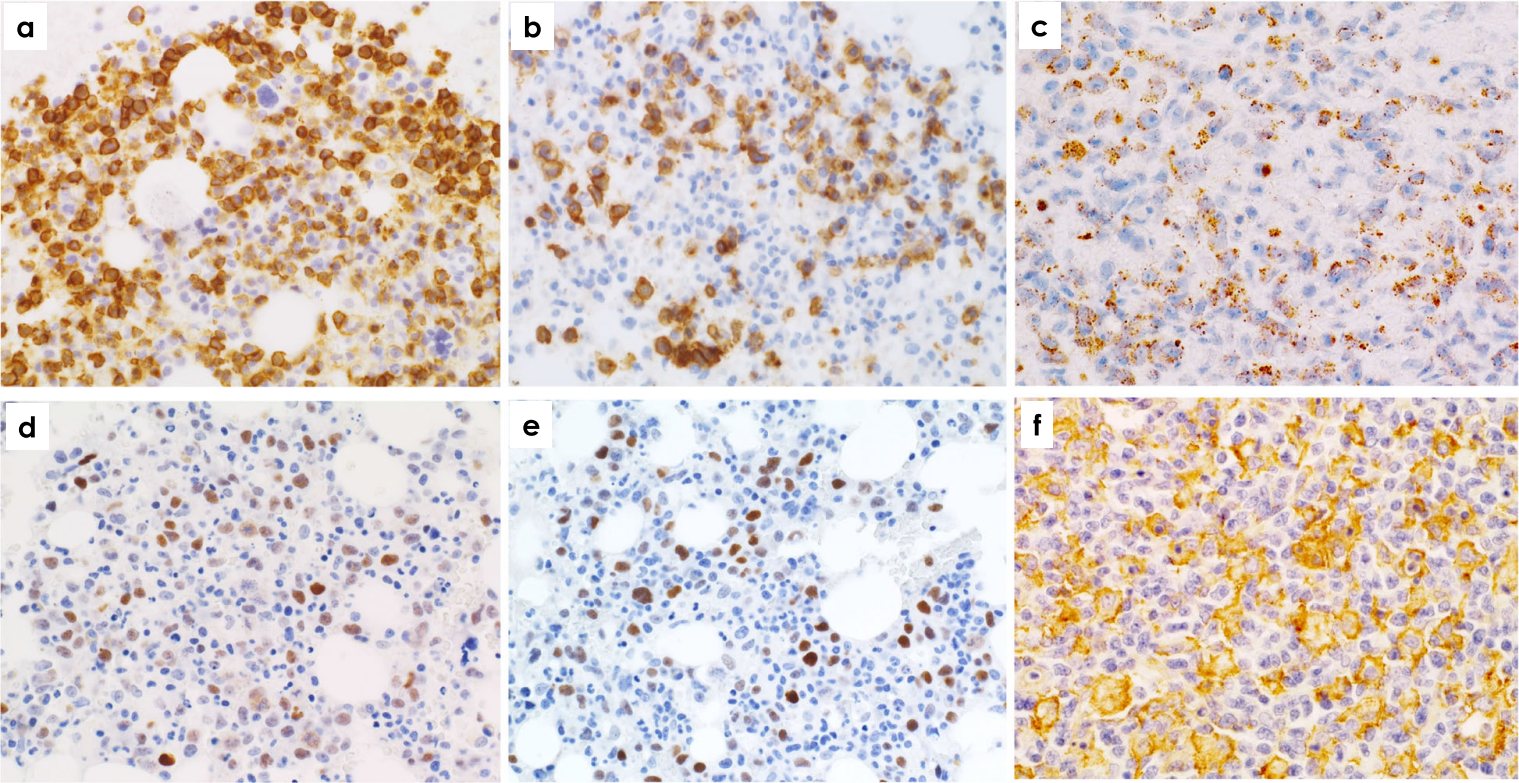
**a** Numerous CD8^+^ medium-sized and large lymphocytes in bone marrow clot sample from patient No. 3. **b** Scattered infiltrate of CD8^+^ medium-sized and large lymphocytes in bone marrow from patient No. 12. **c** Scattered and clustered TIA1^+^ large lymphocytes in hepatic lobules of patient No. 11. **d**, **e** pSTAT3^+^ and CMYC^+^ atypical lymphocytes in bone marrow (patient No. 4). **f** Scattered histiocytes/dendritic cells show strongly positive reaction to PD-L1 (R3^+^) in a lymph node from patient No. 9. × 400

**Fig. 3:**
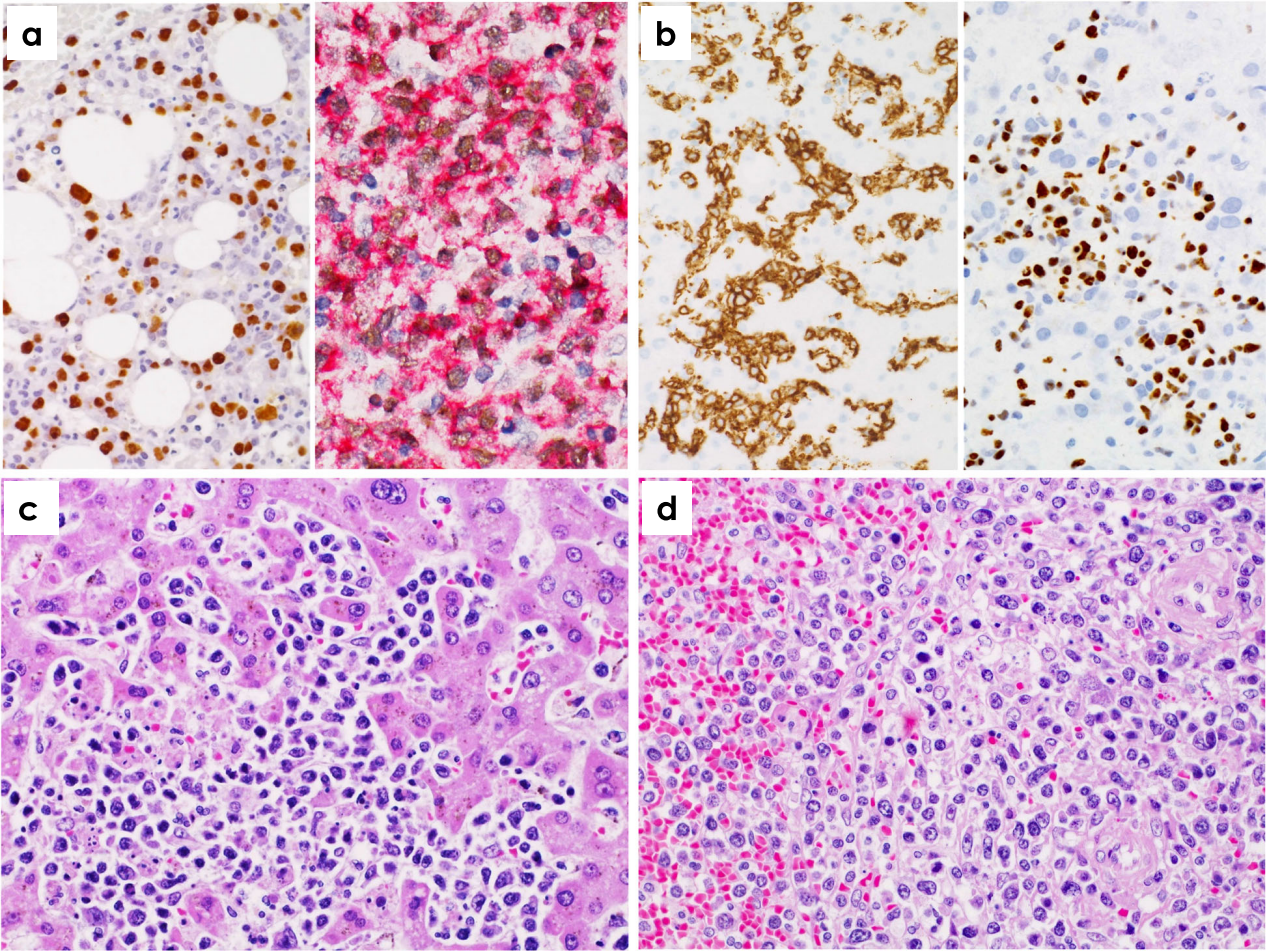
**a** In situ hybridization of Epstein-Barr virus-encoded RNAs (EBERs). Numerous EBERs^+^ transformed lymphocytes detected in bone marrow (left, patient No. 7). Numerous CD8^+^ (red: Fast red) and EBERs^+^ (brown: diaminobenzidine) large lymphocytes observed in a lymph node (right, patient No. 9). **b** Diffuse sinusoidal invasion by CD8^+^ (left) and EBERs^+^ (right) lymphoid cells in liver from patient No. 15. **c** Lobular and focal sinusoidal invasions by large atypical lymphocytes in autopsied liver (patient No. 10). **d** Periarteriolar and partial sinusoidal invasions by large atypical lymphocytes in autopsied spleen (patient No. 11). × 400

Immunohistologically, atypical lymphocytes were diffusely positive for CD3, CD8, and cytotoxic granule proteins in all 16 EBV^+^ TLPD patients (100%) (Fig. 2a, b, c, Fig. 3b), and negative for TCRCγM1, CD4, CCR4, CD20, and CD56. Atypical lymphocytes in the 12 sEBV^+^ TCL patients were positive for CXCR3 in 10 cases (83.3%, Fig. 2d), pSTAT3 in eight (66.7%) (Fig. 2e), CMYC in 10 (83.3%) (Fig. 2f), p53 in nine (75%), PD1 in two (16.7%), and CD30 in one (8.3%). PD-L1^+^ tumor cells (*n* = 1) and strong (R2^+^ and R3^+^) reaction of PD-L1^+^ histiocytes/dendritic cells (*n* = 8) were found in nine sEBV^+^ TCL patients (75%). Two nodal TCL patients showed diffuse infiltrates of CD30^+^, pSTAT3^+^, and CMYC^+^ large lymphocytes. Atypical lymphocytes in two patients with sCAEBV were negative for CD30, CMYC, and p53. Three sEBV^+^ and two EBV^+^ nodal TCL patients had scattered LMP1^+^ and ENBA2^−^ atypical lymphocytes. EBV latency I was found in nine sEBV^+^ TCL patients, and latency II was identified in the remaining three. Many EBERs^+^ atypical lymphocytes were detected in all 16 patients (Fig. 3a, b), and double staining revealed EBERs^+^ nuclear signals in CD3^+^ and CD8^+^ T-cells.

### Examination of the *TCR*γ gene with BIOMED-2 PCR analysis

**Fig. 4:**
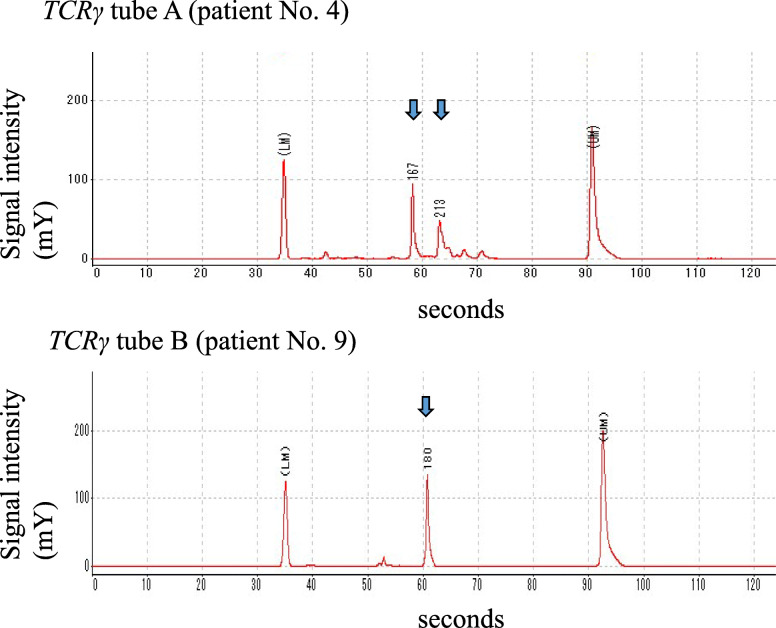
*T-cell receptor (TCR)* γ gene analysis by PCR. Clonal peaks (arrows) of the *TCR*γ gene VJ and DJ regions (Tubes A, B) detected by GeneScan analysis in patient Nos. 4 and 9, respectively. The lower marker (LM) and upper marker (UM) are spiked as internal standards

Using PCR and GeneScan software, examination of the *TCR*γ gene in all eight sEBV^+^ and two EBV^+^ nodal TCL patients revealed clonal peaks of the *TCR*γ VJ and DJ regions (Fig. 4), while one patient with sCAEBV (No. 15) presented polyclonal peaks (Table 2).

### Treatments and prognoses

Combined chemotherapy, including VP16, was administered in six younger and four older sEBV^+^ TCL patients, and allogeneic SCT was performed in three younger patients. Two younger sEBV^+^ TCL patients received steroid pulse therapy because of poor clinical condition. Eight sEBV^+^ TCL patients died of the disease within 4 months, whereas the remaining four younger sEBV^+^ TCL patients who received chemotherapy and allogeneic SCT (in three cases) were alive after 15–48 months. Two EBV^+^ nodal TCL patients received chemotherapy, and one (No. 13) died of the disease in 7 months. One patient with sCAEBV (No. 15) received chemotherapy, but followed a lethal clinical course with liver dysfunction. Another CAEBV patient (No. 16) achieved partial remission in 21 months after receiving dexamethasone and VP16.

### Autopsy examination

In three autopsies of older sEBV^+^ TCL patients, numerous erythrophagocytic macrophages and degenerative atypical lymphocytes with necrosis were detected in bone marrow and systemic lymph nodes. Partly nodular and scattered infiltrates of atypical large atypical lymphoid cells were detected in the lobules, sinuses and portal areas of the liver, and in periateriolar lymphoid sheaths and focally in red pulps of the spleen (Fig. 3c, d). Diffuse and patchy infiltrates of atypical lymphocytes were also detected in lung, pancreas, intestine, and adrenals of two patients.

## Discussion

According to our results, four older patients in 12 sEBV^+^ CD8^+^ TCL showed bone marrow, lymph node, and hepatosplenic invasion of medium-sized to/or large atypical lymphocytes, and exhibited lethal progressive clinical course with HLH. Park et al. reported on fatal five sEBV^+^ TCL patients over 50 years old, who showed bicytopenia (100%), generalized lymphadenopathy (60%), liver dysfunction (80%), rare hemophagocytosis (20%), and no bone marrow invasion (0%) [18]. Among them, three had CD8^+^ small and medium-sized to large atypical lymphocytes. Another recent report showed nine patients with sEBV^+^ TCL predominantly CD8^+^ T cell phenotype, aged between 1 and 46 years old [10]. Our examination of four older patients demonstrated typical clinicopathological features of sEBV^+^ TCL of childhood, and indicated that sEBV^+^ TCL can occur in the elderly.

A prior study of 39 older adults with EBV^+^ nodal cytotoxic TCL showed intermittent bone marrow invasion in 11 patients (29%), thrombocytopenia in 21 (62%), and hemophagocytosis in 13 (38%), and lymphoma cells had CD4^+^ (15%), CD8^+^ (64%), and CD56^+^ (15%) T/NK-cell phenotypes [11]. Median survival of the EBV^+^ nodal TCL patients was 4 months, and thrombocytopenia, bone marrow, and hepatic invasions indicated significantly poor prognostic factors (all, *p* < 0.05). In the present study, two older adults with EBV^+^ nodal CD8^+^ TCL primarily had involvement of systemic lymph nodes without features of HLH. However, one patient (No. 14) had partial bone marrow involvement, bicytopenia, and hyperferritinemia. Although age distribution was different, differences in clinical symptoms between sEBV^+^ CD8^+^ TCL of childhood and EBV^+^ nodal CD8^+^ TCL may be due to the result of differences in the primary involved sites of lymphoma cells.

Coffey et al. reported that six younger patients with sEBV^+^ CD8^+^ TCL showed clinicopathological features similar to CD8^+^ HLH, whereas another two had features similar to sCAEBV with increased atypical CD4^+^ T-cells [19]. EBV^+^ HLH was reported to be primarily characterized by CD8^+^ T-cells with mild nuclear atypia, and roughly 10% of patients followed a progressive clinical course [6, 13]. However, sCAEBV is primarily characterized by small and medium-sized lymphocytes with mild atypia, and CD4^+^ or occasionally CD8^+^ T-cell and CD56^+^ NK-cell phenotypes, and it was reported that three of 13 sCAEBV patients (23.1%) had complicated life-threatening sEBV^+^ TNKCL [20]. Patients with sEBV^+^ CD4^+^ TCL frequently have persistent history of sCAEBV for several months to years before severe manifestations emerge [10, 14, 19]. In the present report, only one older adult with sEBV^+^ CD8^+^ TCL had past history of cutaneous CAEBV-like lesions in 6 months before overt manifestation. Although EBV^+^ HLH, sCAEBV, and sEBV^+^ TCL are difficult to differentiate, and may be serial diseases, the rapidly progressive clinical course, HLH, CD8^+^ atypical lymphocytes with overt nuclear atypia, *TCR* gene rearrangements, and therapy resistance were important findings for making the diagnosis of sEBV^+^ TCL. It is also necessary to confirm the progression mechanism from sCAEBV to sEBV^+^ CD4^+^ and CD8^+^ TCL in younger and older adults.

Patients with sEBV^+^ TCL frequently pursue rapidly progressive clinical course with multiorgan failure. As a treatment strategy, etoposide (VP16) or the HLH-2004 regimen are selected as first-line treatments for sEBV^+^ TCL and HLH, and subsequent allogeneic SCT is chosen for achieving long-term survival [19, 21]. In the current study, only four younger patients with sEBV^+^ CD8^+^ TCL (33.3%) achieved complete remission with the above treatments. The present study demonstrated that patients with sEBV^+^ and EBV^+^ nodal CD8^+^ TCL had frequent expressions of pSTAT3, CMYC, and p53 in infiltrating atypical lymphocytes. Combined PD-L1^+^ tumor cells and strong (R2^+^ and R3^+^) reaction of PD-L1^+^ histiocytes/dendritic cells were frequently found in 12 of 16 patients with EBV^+^ CD8^+^ TLPDs (75%). STAT3 was continuously activated in EBV^+^ T/NK-cell lines and in peripheral blood mononuclear cells from CAEBV patients [22]. Furthermore, ruxolitinib (JAK1/2-STAT3 inhibitor) suppressed the phosphorylation of STAT3 and decreased the number of viable cells. PD-L1^+^ tumor and non-neoplastic cells in EBV^+^ nodal CD8^+^ TCL were more frequently observed with immunohistology and multiplex immunofluorescence than in extranodal NK-cell lymphoma [23]. These protein expression patterns may be effective to promptly recognize the severity of the diseases. Furthermore, studies of the cell characteristics in EBV^+^ CD8^+^ TLPDs are necessary to select additional target therapies for preventing a lethal clinical course.

In conclusion, we mainly presented 12 patients with sEBV^+^ CD8^+^ TCL, four of whom were over 60 years old. Only one older adult had past history of cutaneous CAEBV-like lesions. sEBV^+^ CD8^+^ TCL was characterized by medium-sized to/or large atypical lymphocytes, primarily in bone marrow, lymph nodes, liver, and spleen, with features of HLH, and rapidly progressive clinical course. sEBV^+^ CD8^+^ TCL was frequently positive for pSTAT3, CMYC, and p53, which may be effective to recognize the severity of the diseases. Strong (R2^+^ and R3^+^) reactions of PD-L1^+^ histiocytes/dendritic cells were frequently found in patients with sEBV^+^ CD8^+^ TCL. Four younger patients of the 12 sEBV^+^ TCL (33.3%) are alive with chemotherapy including etoposide, three of whom underwent allogeneic SCT. Additional target therapies are necessary to prevent a lethal clinical course of EBV^+^ CD8^+^ TLPDs.

## Abbreviations

CAEBV: Chronic active EBV infection
CXCR3: Chemokine receptor 3
EBV: Epstein-Barr virus
EBERs: EBV-encoded RNA
HLH: Hemophagocytic lymphohistiocytosis
IM: Infectious mononucleosis
NK: Natural killer
PD1: Programmed cell death 1
PD-L1: Programmed cell death-ligand 1
pSTAT3: phosphate signal transducer and activator of transcription
SCT: Stem cell transplantation
TCL: T-cell lymphoma
TCR: T-cell receptor
TLPDs: T-lymphoproliferative diseases
